# Supplementary material for: Cognitive reserve against Alzheimer’s pathology is linked to brain activity during memory formation
Source: Nat Commun. 2024 Nov 13;15:9815. doi: 10.1038/s41467-024-53360-9 (PMC11561234; doi:10.1038/s41467-024-53360-9)
Supplement: Supplementary file 1 — Supplementary Material [file 41467_2024_53360_MOESM1_ESM.pdf]

# 5 Supplementary Material

## 5.1 Supplementary Methods

### 5.1.1 MRI acquisition and processing

The supplementary analyses made use of two additional MRI modalities that have been acquired: T2-weighted FLAIR images (3D GRAPPA PAT 2, 1mm$^3$ isotropic, 256x256 px, 192 sagittal slices, TR 5000ms, TE 394ms, TI 1800ms, ca. 7min) and resting-state fMRI data (2D EPI, GRAPPA PAT 2, 3.5mm$^3$ isotropic, 64x64 px, 47 slices, oblique axial/AC-PC aligned, TR 2580ms, TE 30ms, FA 80°, 180 volumes with interleaved acquisition, ca. 8 min).

White matter hyperintensity (WMH) probability maps were obtained from FLAIR scans with the Lesion Prediction Algorithm in the Lesion Segmentation Toolbox.[1] These maps underwent thresholding with a value of 0.2, and the total WMH volume was determined by tallying the number of voxels that remained after the thresholding process. To account for intracranial differences, fractional WMH volumes were computed (i.e. total WMH volume divided by TIV).

Resting-state fMRI data processing and analysis were done with the CONN Toolbox, SPM12 and Matlab_R2022a. Preprocessing in CONN included realignment and unwarping and slice time correction. Prior to further preprocessing $\text{FD}_{Jenk}$ was calculated as the root mean squared volume-to-volume displacement of all brain voxels measured from the six head motion parameters[2] and used to exclude outlier subjects based on the following criteria adapted from Parkes et al.[3]: if mean $\text{FD}_{Jenk}$ was greater than 0.25 mm, if $\text{FD}_{Jenk}$ of any EPI was more than 5 mm, or if 33% of all the 180 EPIs had $\text{FD}_{Jenk}$ above 0.25mm. After coregistration and normalization to a study-specific template space (created via Shoot in SPM), a customized denoising pipeline in CONN implemented an anatomical component-based noise correction procedure. It comprised five noise components from white matter and CSF,[4] estimated subject-motion parameters using Friston-24 parameter model,[5] identified outlier volumes ($\text{FD}_{Jenk}$ $> 0.25$ mm), and global signal regression. Temporal band-pass filtering (0.01-0.08 Hz) was additionally applied to eliminate the effect of physiological noise after regression as recommended.[6] Subsequently, resting-state functional connectivity within seven standard networks (see Yeo et al.[7]) was obtained using CONN's second-level analysis.

### 5.1.2 Quality control and sample cleaning

As another means to improve signal to noise ratio we chose strict criteria for outlier exclusion based on behavioral and task fMRI metrics. Individuals were excluded if either of the following was true: (1) They made more than 8 errors in their indoor/outdoor judgment. This corresponds to individuals with extreme outliers in the distribution of indoor/outdoor errors and could be related to lack of attention or confusion. (2) Based on response bias in their confidence rating during post-MRI retrieval, represented by the criterion location $c = -\frac{1}{2}(\text{z(HR)} + \text{z(FAR)})$; z = normal inverse cumulative distribution function,

HR = hit rate, FAR = false alarm rate. Individuals with absolute response bias values above 1.5 were excluded, since strong bias could potentially render the parametric modulation invalid for two reasons. First, the response category would likely not correspond to the actual BOLD signal at the time of encoding. Second, a reliable estimation of the subsequent memory regressor does require some variability in the response categories. (3) Framewise displacement (FD) was above 0.5mm in a single EPI or above 0.2mm in more than 2% of the EPIs. This exclusion was supposed to limit motion effects on the data quality. (4) An individual had extreme outliers in the $\beta$ values of more than 10% of the voxels of their (GM-masked) regressor image. This was indicative of inaccurate estimations of the subsequent memory regressor in large parts of the brain and could have skewed the results of subsequent modeling steps. 68 individuals (11 CN, 6 ADR, 20 SCD, 19 aMCI, 12 AD dementia) were excluded based on these criteria, leaving an fMRI sample with 490 individuals.

### 5.1.3 One-dimensional pathological load score

Due to the nonlinearity of the disease progression trajectory along the AD continuum in 3D ATN space ($A\beta_{42:40}$ratio, CSF p-tau, hippocampal volumes), a nonlinear dimensionality reduction method called t-SNE [8] was employed to reduce the dimension to one, yielding a single PL score per subject. Broadly speaking, t-SNE converts the Euclidian distances between datapoints in the high-dimensional space into conditional probabilities, which represent similarities. Likewise, conditional probabilities are defined for the low-dimensional counterparts. A perfect representation of the data in a lower-dimensional space would retain the conditional probabilities from the high-dimensional space between all pairs of datapoints. Hence, an optimal solution is sought by minimizing the mismatch between the conditional probabilities in both spaces, which is quantified via the Kullback-Leibler divergence. More details about the method can be found in Van der Maaten and Hinton [8]. Assuming that the biomarker progression profile across individuals is homogeneous, t-SNE would be able to extract this progression profile faithfully by retaining the similarities between datapoints from the ATN space as much as possible in the one-dimensional output space.

As the name suggests, t-SNE is a stochastic algorithm. Hence, the random seed was fixed to 617 to ensure reproducibility. The algorithm was applied to all 441 participants with complete ATN data available using a perplexity parameter of 50. One individual was considered an outlier and removed from all analyses, as visual inspection indicated that its assigned PL score was an erroneous representation of its AD biomarker status, as assessed from comparison to all other individuals' PL scores (see Fig. S4 for a plot including the declared outlier). Subsequently, the resulting score was scaled to fall into the range between 0 and 1 by subtracting the minimal value across participants and then dividing it by the maximal value. For reasons of interpretability, the scale was reversed such that increasing numbers of the PL score refer to increasing amounts of AD pathology.

Moreover, the algorithm was tested with five different choices of the perplexity parameter (10, 25, 30, 50, 100) and the results were correlated with each other to check stability. On average, the correlation was 0.950, indicating that the obtained score does not exhibit substantial dependence on the selected perplexity parameter. Robustness of the PL score was further checked by applying the t-SNE algorithm 1000 times, each time randomly holding out 10% of the data. The mean correlation over 1000 iterations was 0.945, suggesting a high robustness of the proposed PL score based on ATN.

#### 5.1.4 Cross-validation to determine optimal number of principal components

The optimal number of principal components $P$ was determined in a 10-fold cross-validation approach. Its results are shown in Fig. S8. Since not all participants with functional data also had CSF measures and thus a PL score, the data was stratified into two groups accordingly. This ensured similar proportions of both groups within each fold, as participants with missing values for any of the variables in the moderation model could still be used for principal component analysis (PCA). PCA was performed separately on the training data in each of the folds after mean-centering the masked functional (training) data. The coefficients of the moderation model from Eq. 2 were then determined for different numbers of principal components (1-25) using the least-squares method. With these coefficients the held-out (test) data was predicted. Across the ten folds, all data was predicted once based on the remaining 90% for each number of principal components. The coefficient of determination ($R^2$) between the true and predicted PACC5 values (Box-Cox transformed) was calculated based on the aggregated data, done once per number of principal components. In order to ensure independence of a particular division into folds, this procedure was repeated 10 times with different partitioning of the data into folds. The optimal number of principal components was identified as the corresponding model with the highest mean $R^2$ value across the 10 predictions.

#### 5.1.5 Alternative bootstrapping for voxel-wise inference

An alternative approach for voxel-wise inference via bootstrapping was inspired by Chen et al.[9] who presented it in the context of multivariate mediation. It has been slightly adjusted for the multivariate moderation model. In contrast to the employed inference approach presented in the main text, which considers distribution of individual voxels to determine their significance, this approach considers a global distribution that is assembled as follows. Again, it starts with 5000 iterations of bootstrapping:

1. Create a bootstrap sample of equal size as the original sample used in the multivariate moderation model by randomly resampling subjects with replacement from it.
2. Estimate bootstrap coefficients $\hat{b}_{3,p}$ from Eq. 2 for the bootstrap sample.

3. Obtain individual voxel bootstrap moderation coefficients $\hat{w}_i$.

The coefficients $\hat{w}_j$ were then stacked into a 5000*13695 matrix. Subsequently, the bootstrap distribution of all voxels was sorted by their median values and voxels whose median lies within the second or third quartile were selected. 10 % of all coefficients $\hat{w}_j$ from the selected voxels were randomly sampled and combined to form a pseudo-null distribution. Finally, the pseudo-null distribution was utilized to fit a normal distribution, which can be used to obtain a p value for every voxel based on its coefficient $w_i$. Voxels with p values below 0.05 were regarded as significant. Please note that non-identifiability of the sign of the coefficients is, unlike in the multivariate mediation approach, not a problem in the multivariate moderation approach and thus the inference step presented here deviates slightly from the one in Chen et al.[9].

This approach had been utilized in a previous version of the paper, but was changed for the current, more naive approach for multiple reasons. First, it allowed us to quantify uncertainty in the estimations of the voxel-wise CR weights (used to obtain confidence intervals in Fig. 5). Second, when considering a multivariate model with 2 instead of 7 PCs, the distribution of CR weights was not unimodal normal, but rather bimodal and slightly non-normal (see Fig. S11E), making it insensible to fit a normal distribution for inference. Third, the assessment of local distributions instead of a global distribution seemed more sensible with the data. Significant brain regions according to this inference approach are shown in Fig. S12 for a multivariate model with 7 or 2 PCs, respectively.

## 5.2 Supplementary Results

### 5.2.1 Construction of a continuous one-dimensional pathological load score

Based on the ATN framework that represents a biological characterization of AD,[10] a novel data-driven index for disease severity along the AD continuum was constructed combining CSF and MRI biomarkers of ATN in a one-dimensional score. The score ranges from 0 to 1, where 0 represents minimal AD pathology and 1 means maximal AD pathology in reference to the underlying sample. As demonstrated in Fig. S1, the underlying continuum follows a nonlinear pattern capturing dependencies across all three ATN biomarkers. More specifically, low $A\beta_{42:40}$ ratios (A), high p-tau measures (T), and small hippocampal volumes (N) result in high PL scores. Figs. S1A and C suggest that $A\beta_{42:40}$ is generally the strongest contributor to the PL score.

In the lowest range of PL scores from 0 to about 0.2, lower $A\beta_{42:40}$ratios seem to be the main determinant of the PL score, which is thought to represent disease severity (Fig. S1). From roughly 0.2 until 0.5, higher values of the PL score are mainly characterized by lower hippocampal volumes (Fig. S1F). From there until PL scores of ca. 0.75 it is again primarily lower $A\beta_{42:40}$ ratios that contribute to a higher PL score. On the upper end of the PL score above 0.8, p-tau is the main dimension of variance (Fig. S1E).

Additionally, the correlation between the PL score and its single components was tested, to examine which dimension is (linearly) most strongly related to the PL score. The cross-correlations are -0.862 for $A\beta_{42:40}$, 0.670 for p-tau

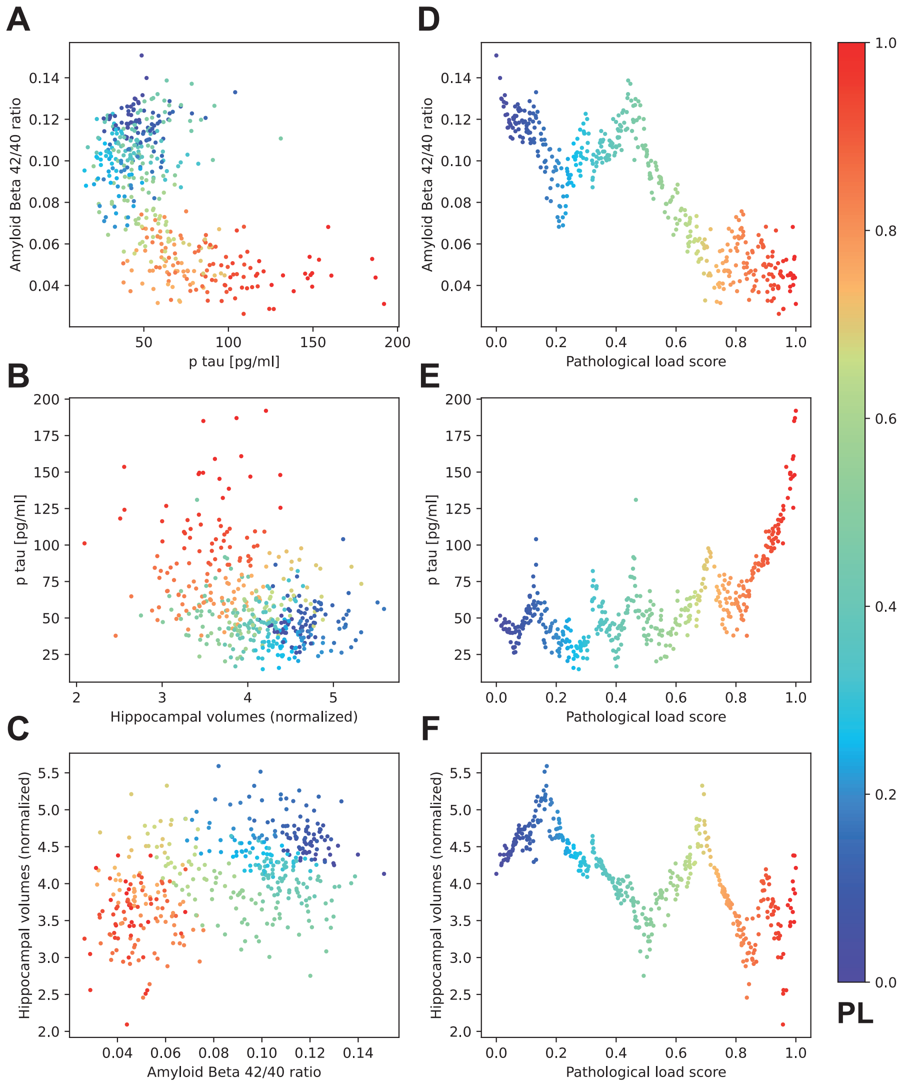


Figure S1: **Relationship between ATN biomarkers and data-driven PL score.** (A-C) All three possible combinations of pairs of biomarkers are shown. The points are color-coded based on the corresponding pathological load score. (D-F) Biomarkers plotted against the PL score. Hippocampal volumes refer to bilateral hippocampal volumes corrected for TIV. Please note that coloring of data points is redundant in panels D-F and was only done for illustrative purposes. Source data are provided as a Source Data file. PL = pathological load, TIV = total intracranial volume.

and $-0.684$ for the TIV-corrected hippocampal volumes. This confirms the impression that the A dimension had the biggest influence on the construction of the PL score. P-tau and the hippocampal volumes are reflected in the PL score to a similar extent, with different signs. This matches the observation that AD severity is associated with higher p-tau measures and lower hippocampal volumes.

In summary, the substantial correlations of the PL score with the ATN measures suggest shared variance and hence a meaningful reduction of the AD continuum to a single dimension.

### 5.2.2 Pathological load is associated with cognitive performance

As a marker for disease severity on the AD continuum, the PL score was expected to show strong associations with the cognitive measures from neuropsychological testing. Indeed, we found a strong association between PL and PACC5 as measure of cognitive performance ($p = 2.00 \cdot 10^{-22}$, standardized regression coefficient $\beta$ = -0.469 [-0.559,-0.380], t(400) = -10.356). However, the empirical associations suggested rather a nonlinear relation, i.e. an increasing rate of cognitive decline with increasing pathology (Fig. 1). Hence, we further tested whether cognitive performance follows a quadratic function of the PL score. The association between (quadratic) PL and cognitive performance was significant ($p = 5.03 \cdot 10^{-28}$, $\beta$ = -0.516 [-0.602,-0.431], t(400) = -11.864). In concordance with the differences in $\beta$, a comparison of the $R^2$ values revealed that the purely quadratic model fits the data better ($R^2 = 0.385$) than the linear model ($R^2 = 0.345$).

### 5.2.3 Longitudinal validation model

In addition to the longitudinal model presented in the main text, which included hippocampal atrophy to represent pathology and was available for 485 participants with a total of 1544 observations, a similar LME was created for the subsample with PL scores (722 observations for 229 participants). The model showed an interaction of pathology with time (p = 0.006, $\beta$ = -0.089 [-0.156,-0.026], t = -2.795, df $\approx$ 87.6), a three-way interaction of the CR score with PL and time ($p = 1.46 \cdot 10^{-4}$, $\beta$ = 0.144 [0.073,0.217], t = 3.990, df $\approx$ 79.6) and a simple interaction of the CR score with pathology ($p = 4.35 \cdot 10^{-11}$, $\beta$ = 0.420 [0.300,0.539], t = 6.929, df $\approx$ 226.5).

Additionally, the longitudinal model of the main text was fit in the subsample without PL scores, i.e. the participants that the multivariate moderation model had not seen during training (822 observations for 256 participants in the LME). The results were weaker but consistent with the results in the whole sample. There was again an interaction of pathology with time ($p = 6.60 \cdot 10^{-7}$, $\beta$ = -0.182 [-0.255,-0.114], t = -5.314, df $\approx$ 992), a statistical trend for the three-way interaction of the CR score with PL and time (p = 0.057, $\beta$ = 0.083 [-0.003,0.170], t = 1.925, df $\approx$ 89.3) and a simple interaction of the CR score with pathology ($p = 1.77 \cdot 10^{-4}$, $\beta$ = 0.271 [0.131,0.411], t = 3.805, df $\approx$ 258.7).

### 5.2.4 Association of the CR score with other variables

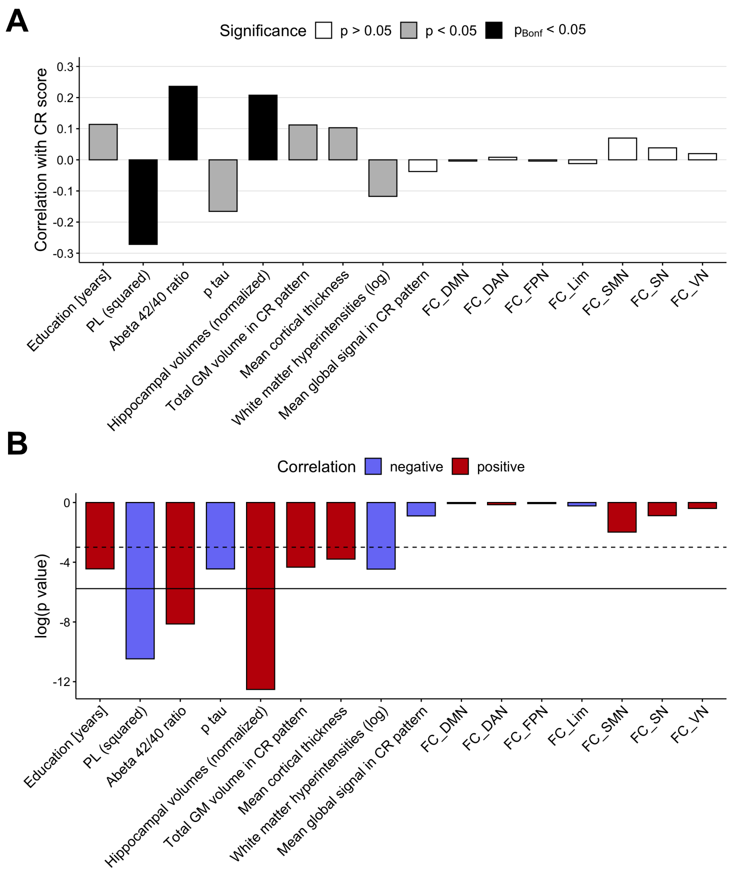


Figure S2: **Correlation of CR score with other variables.** (A) Pearson correlation coefficients between the CR score and the other variables. (B) P values for the corresponding correlations of panel A, illustrated on a logarithmic scale. Bars below the dashed line have $p < 0.05$, below the solid line a Bonferroni-corrected $p < 0.05$. Functional connectivity (FC) measures came from resting-state data. Mean global signal refers to task fMRI. Please note that the sample sizes differed between comparisons: n = 231 for CSF(-related) measures, n = 452 for all FC measures, n = 462 for white matter hyperintensities, n = 489 for the other variables. CR = cognitive reserve, DMN = default mode network, DAN = dorsal attention network, FPN = fronto-parietal network, GM = gray matter, Lim = limbic network, PL = pathological load, SMN = somatomotor network, SN = salience network, VN = visual network.

In an attempt to understand why certain participants are able to maintain activation patterns, we investigated potential predictors and contributors of the CR score. In this sample, neither resting-state functional connectivity within seven standard networks nor mean global task-fMRI signal were significantly related to CR score variability (see Fig. S2). However, higher CR scores were as-

sociated with less pathological measures of AD burden such as the (squared) PL score ($p = 2.84 \cdot 10^{-5}$, r(229) = -0.272 [-0.387,-0.148]), its components $A\beta_{42:40}$ ($p = 2.93 \cdot 10^{-4}$, r(229) = 0.189 [0.110,0.354]), p-tau (p = 0.017, r(229) = -0.166 [-0.289,-0.037]) and hippocampal volume ($p = 3.65 \cdot 10^{-6}$, r(487) = 0.208 [0.121,0.291]), as well as lower global white matter hyperintensity volumes (p = 0.035, r(460) = -0.098 [-0.188,-0.007]). The CR score was further weakly positively correlated with total GM volumes in the regions with significant contributions to CR (p = 0.013, r(487) = 0.112 [0.024,0.199]) and mean cortical thickness (p = 0.023, r(487) = 0.103 [0.015,0.190]). Yet, it should be noted that accounting for these structural differences as covariates had essentially no effect on the observed results, neither in the multivariate moderation model nor the validation analyses of the CR score (see Fig. S19) and Tab. S1. Taken together, although AD pathology indices, tissue volumes and white matter lesions were slightly associated with individual CR score differences, the pattern of CR-related brain areas and its predictive value for memory performance was not mediated by atrophy alone or network connectivity at rest.

Due to the relationship between PL and the CR score, we also tested a formal mediation, which indicated that the CR score mediates the effect of PL (squared) on PACC5 (average causal mediation effect: -0.1566 [-0.3229, -0.0300], p = 0.013).

#### 5.2.5 Validation models for alternative CR scores

To study the robustness of the model, we recalculated the findings with a model of significantly reduced complexity. When fitting a multivariate moderation model with only 2 instead of 7 principal components for the functional data (see Fig. S11) and deriving a new CR score based on the coefficients, the corresponding validation models consistently indicated worse predictions of cognitive performance compared to models with the CR score (based on 7 PCs) presented in the main text. One exception was an analysis involving the domain-general cognitive factor in the MRI-only sample. For the cross-sectional analyses in the sample with available PL scores, the $R^2$ values for the models with the interaction between PL and CR score on cognition were 0.472 for PACC5, 0.486 for the latent memory factor (f_mem) and 0.416 for the global cognitive factor (f_glob), in contrast to $R^2$ values of 0.534, 0.533 and 0.476, respectively, for the CR score based on 7 PCs. In the MRI-only sample with hippocampal atrophy instead of PL scores, the difference in $R^2$ between 2 versus 7 PCs was less dramatic for PACC5 and f_mem ($R^2$ = 0.425 for PACC5, 0.442 for f_mem as compared to 0.441 for PACC5, 0.443 for f_mem when using 7 PCs). $R^2$ for f_glob was slightly higher with 0.363 when using 2 PCs in comparison to 0.353 when using 7 PCs. In the longitudinal model with the three-way interaction of hippocampal atrophy with the CR score and time, the AIC was 2289.2 for a CR score based on 2 PCs as compared to 2267.5 for the CR score based on 7 PCs (reported in the main text), once again indicating a worse model fit with similar interpretations for the three-way interaction. In summary, the validation analyses suggest that a CR score based on a multivariate moderation model in which the functional

patterns are described using only 2 principal components instead of 7 is worse at explaining the cognitive performance data, possibly increasing the risk for bias (underfitting). This indicates that two components are likely to be insufficient to adequately represent cognitive reserve patterns in its complexity. On top of that, seven components were superior to any other number of principal components according to the cross-validation.

### 5.2.6 Inclusion of structural covariates in validation models

As seen in the previous section, there was a weak correlation of the CR score with structural measures, namely mean cortical thickness (obtained from Freesurfer) and mean gray matter volumes (from segmentation with SPM) in the voxels with significant contributions to CR. To ensure that the CR score represents reserve beyond mere structural integrity, we added a corresponding covariate to the multivariate moderation model. The results were found to be essentially the same as before, with correlations of 0.999 for mean cortical thickness and 0.995 for the mean GM volumes (see Fig. S19). Furthermore, we performed additional analyses analogous to the validation analyses in the main text, but with an additional morphometric covariate. Once again, the results were very similar, with only minor differences in the p values of the interaction effect of the CR score (see Tab. S1).

Table S1: Results of the models with the CR score. Shown are the p values of the interaction effects of the CR score with pathology (either PL or hippocampal atrophy) on cognitive performance (PACC5/latent memory factor/latent global cognitive factor). Column one indicates which structural covariate has been used; no struct cov = original models, without structural covariates; mean cth = mean cortical thickness (Freesurfer); mean GM vol = mean gray matter volume (SPM) of voxels with significant contribution to CR. In the longitudinal model, the p value refers to the triple interaction of the CR score with time and hippocampal atrophy. The p values were calculated from two-tailed t tests of the regression coefficients (degrees of freedom of the longitudinal models were implicitly estimated via Satterthwaite's degrees of freedom method with the lmertest package in R) and not corrected for multiple comparisons. CR = cognitive reserve, PACC5 = Preclinical Alzheimer's Cognitive Composite 5, PL = pathological load.

| | Cross-sectional | | | | | | Longitudinal |
|---|---|---|---|---|---|---|---|
| | PL | | | Hipp. volumes | | | |
| | PACC5 | F_mem | F_glob | PACC5 | F_mem | F_glob | PACC5 |
| no struct cov | $9.15\cdot10^{-15}$ | $8.38\cdot10^{-12}$ | $2.15\cdot10^{-8}$ | $2.35\cdot10^{-6}$ | $2.69\cdot10^{-4}$ | 0.020 | $1.19\cdot10^{-4}$ |
| mean cth | $1.42\cdot10^{-14}$ | $1.20\cdot10^{-11}$ | $2.88\cdot10^{-8}$ | $1.18\cdot10^{-6}$ | $1.49\cdot10^{-4}$ | 0.015 | $1.30\cdot10^{-4}$ |
| mean GM vol | $1.06\cdot10^{-13}$ | $6.18\cdot10^{-11}$ | $1.57\cdot10^{-7}$ | $3.85\cdot10^{-6}$ | $5.87\cdot10^{-4}$ | 0.049 | $1.26\cdot10^{-5}$ |

An alternative approach to creating a CR score, which is not based on the consensus framework, was to quantify the similarity of the individuals' brain activity during successful memory encoding with the average activity of the cognitively normal individuals (similar to previous approaches like the FADE[11] or FADE-SAME scores[12]). Hence, a similarity score was calculated as a t-weighted sum of the individuals' parametric SM contrast with the group-level t contrast of the cognitively normal individuals. Indeed, the similarity score was correlated substantially with the CR score (r = 0.556). The results in the

validation analyses were also similar, although the models with a similarity score instead of the CR score explained slightly less variance in the cognitive outcomes. For the cross-sectional analyses in the sample with PL scores, the $R^2$ values were 0.460 for PACC5, 0.503 for the latent memory factor and 0.422 for the global cognitive factor when using the t-weighted sum score (0.534, 0.533 and 0.476, respectively, for the CR score). In the MRI-only sample with hippocampal atrophy instead of PL scores, $R^2$ values were lower for the t-weighted sum score ($R^2$ = 0.411 for PACC5, 0.426 for f_mem, 0.351 for f_glob as compared to $R^2$ = 0.441, 0.443, 0.353, respectively). For the domain-general cognitive score, the p value of the interaction of the similarity score with hippocampal atrophy was not significant ($p = 0.277$). In the longitudinal model, the AIC was higher, i.e. worse, when using the t-weighted sum score (2304.9 compared to 2267.5 for the original CR score). While this is noteworthy, the disadvantages of the similarity score compared to the proposed explicit approach are briefly addressed in the discussion of the main text.

## 5.3 Supplementary Discussion

### 5.3.1 PL score

A cautionary note should be made about the PL score. One should be aware that it is a purely cross-sectional construct that is agnostic for the order of events along the disease progression towards Alzheimer's disease. It has to be stressed that biomarker levels at progressing PL scores hence cannot be interpreted as a sequence of events. In fact, according to the prevailing model of the Alzheimer's pathological cascade, abnormal levels of Aβ are the initiating event in AD,[13] followed by abnormal tau levels and neurodegeneration. However, we found that hippocampal volumes already showed a strong contribution to increasing PL scores at its lower levels, which would indicate atrophy as an early pathological change.

The challenges for a dimensionality reduction method in this context are many-fold. The t-SNE method used here tries to maintain neighborhood relationships between data points in lower-dimensional spaces. In a three-dimensional space, one could potentially find four data points that are mutually equidistant. There is no way to accurately retain these distances in a one-dimensional space. This is part of a challenge known as the crowding problem.[8] Given the composition of the sample, which has an over-representation of individuals devoid of even early clinical symptoms, more severe AD biomarker profiles might not be represented faithfully in the PL score, as the algorithm tries to the preserve the distances beween the many less progressive AD biomarker profiles. On the other side, also age-related and other non-AD related pathological changes (e.g. hippocampal scleroris or vascular disease) might contribute to hippocampal atrophy, neurodegeneration and cognitive decline in our sample. This might be responsible for the stronger contribution of hippocampal volumes to lower PL scores. Furthermore, the influence of uncaptured covariates should not be underestimated. However, even if the sample uniformly represented individuals

across different stages of the AD continuum, recent evidence for distinguishable AD subtypes (see e.g. Vogel et al.[14]) indicate the potential presence of multiple differential biomarker trajectories. In concert, these challenges might explain the unexpected pattern of trajectory reversals of the biomarkers with increasing PL scores that can be observed in Fig. S1.

Nevertheless, for the aim of examining cognitive reserve we make the simplified assumption that AD biomarkers can actually be represented by a single variable. The strong associations with the three biomarkers as well as with cognitive performance indeed suggest the PL score as a meaningful index of overall disease severity (rather than disease progression) for our purpose.

## 5.4 Supplementary Figures

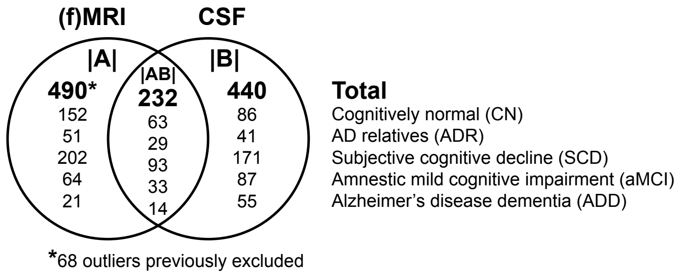


Figure S3: **Venn diagram of the sample**. Sample |A| refers to the participants with fMRI data and was used to derive eigen-images of the subsequent memory contrast images via principal component analysis. Sample |B| refers to the participants with CSF data, which was used for creating the PL score. Sample |AB| is the union of both, i.e., the participants with both fMRI and CSF data. Sample |AB| was used to derive the CR-related activity patterns. The remaining participants of sample |B| that were not part of |AB| were used for further validation of the CR score by determining its ability to moderate the effect of neurodegeneration on individual measures of cognitive performance. AD = Alzheimer's disease, CR = cognitive reserve.

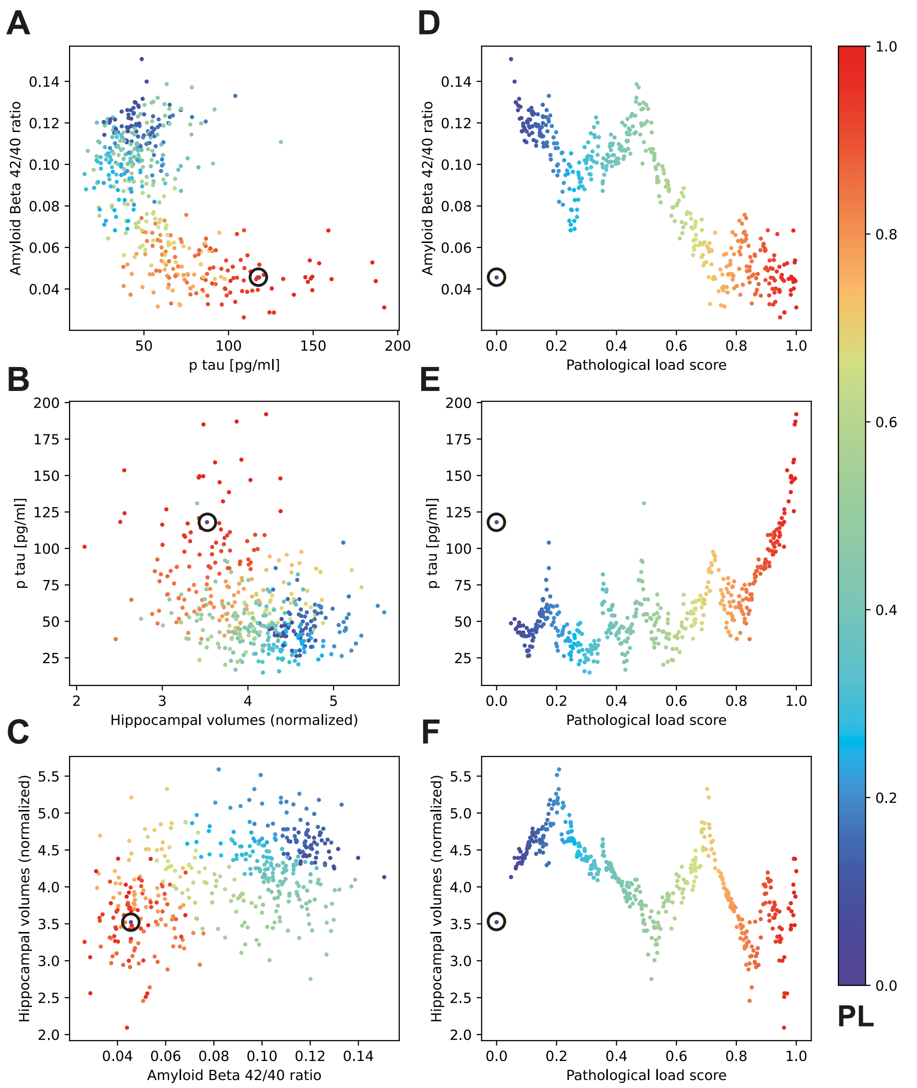


Figure S4: **Relationship between ATN biomarkers and data-driven PL score.** Same as Fig. S1, with the exception that one additional subject was included in the derivation of the PL score, which was deemed an outlier and excluded from all analyses. It is marked with a black circle and has a PL score of 0 despite its very low $A\beta_{42:40}$ratio of 0.46, rather high p-tau levels of 118 pg/ml and TIV-normalized hippocampal volumes of 3.52. (A-C) All three possible combinations of pairs of biomarkers are shown. The points are color-coded based on the corresponding pathological load score. (D-F) Biomarkers plotted against the PL score. Source data are provided as a Source Data file. PL = pathological load, TIV = total intracranial volume.

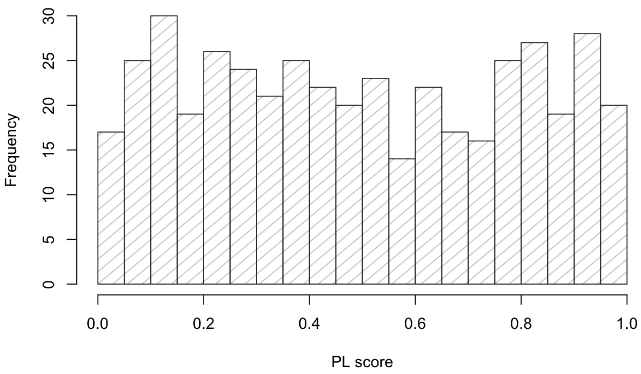


Figure S5: **Distribution of PL score**. The displayed sample is the full CSF sample (|B| in Fig. S3). Source data are provided as a Source Data file. PL = pathological load.

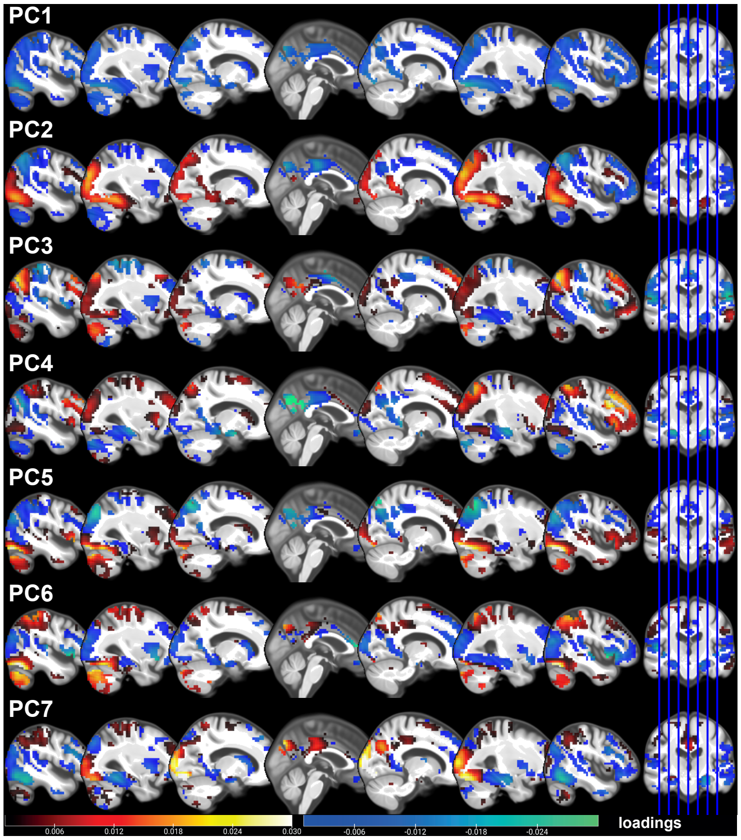


Figure S6: **Principal components of subsequent memory regressor images**. Shown are the loadings of each voxel onto each of the seven principal components (PC1-7) of the regressor images of subsequent memory. Please note that the principal component analysis has been restricted to regions with a significant subsequent memory effect in the baseline sample (compare Fig. 2A) and hence does not include region outside of those 13695 voxels. Source data are provided as a Source Data file.

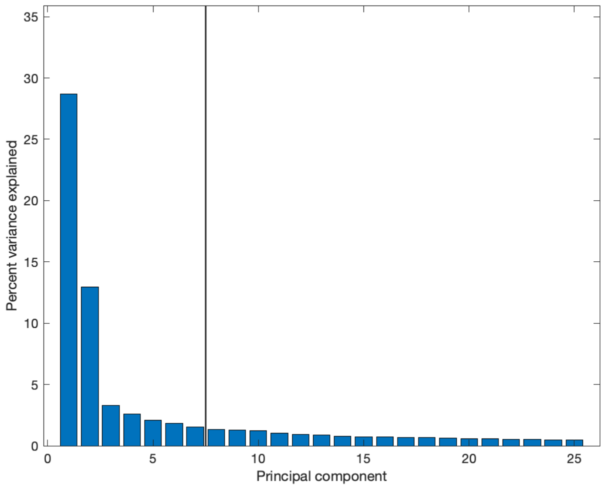


Figure S7: **Variance explained by principal component (eigen-)images**. Shown is the amount of variance in the original subsequent memory regressor images that the individual principal components explain as assessed via their eigenvalues. The black line symbolizes the optimal number of principal components determined via a 10-fold cross-validation procedure. Source data are provided as a Source Data file.

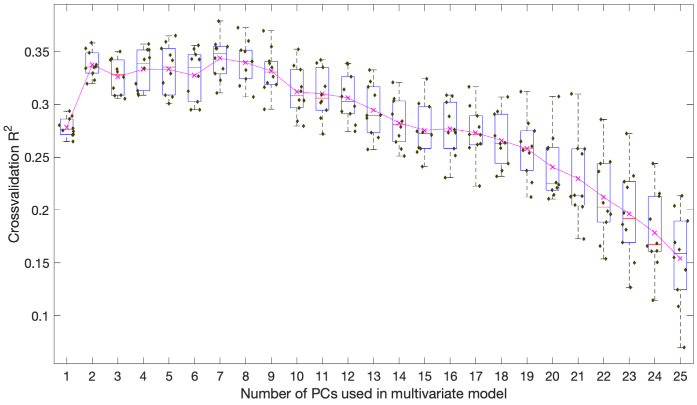


Figure S8: **Cross-validation results**. According to Eq. 2, PACC5 was predicted by varying numbers of principal components (eigen-images) in a 10-fold cross-validation procedure that was repeated 10 times with different partitioning of the data (see section 4.7 for details). The boxplots refer to the cross-validation $R^2$ (coefficient of determination) in PACC5 scores (Box-Cox transformed) in the 10 independent test set predictions. The line shows the median, the box limits the upper and lower quartiles, respectively, and the whiskers have a maximum length of 1.5*IQR. There were no outliers. The magenta crosses (connected via lines) denote the mean values across the 10 predictions and the olive-colored diamonds the individual coefficients of determination. 7 principal components achieved the best cross-validation results. Source data are provided as a Source Data file. IQR = interquartile range, PACC5 = Preclinical Alzheimer's Cognitive Composite 5.

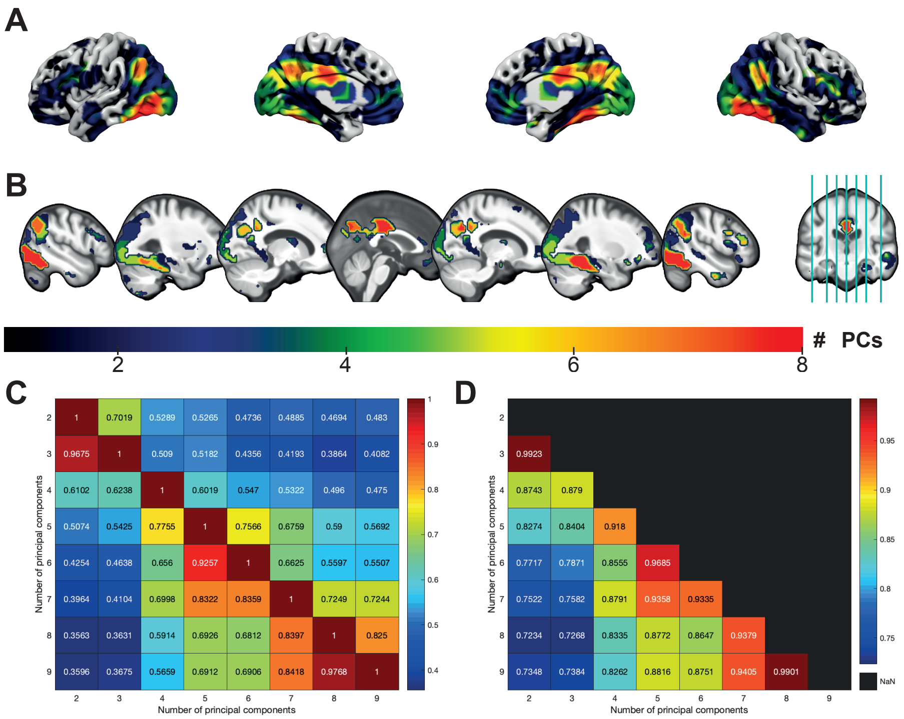


Figure S9: **Significant CR regions across different numbers of principal components.** Like in the cross-validation procedure (Fig. S8), the multivariate model has been fitted on the whole dataset using different amounts of principal components, ranging from 2 to 9. Subsequently, the corresponding voxelwise moderation effects (CR weights), voxels with significant CR weights and individual CR scores have been determined. (A) Surface plot of significant regions. (B) Same as A, but as slice views. Color bar indicates how many of the 8 analyses determined a certain voxel as significant contributor to CR. (C) Heat maps comparing the results in dependence of the number of principal components used in the multivariate model based on the correlation of the voxelwise CR weights (lower triangular part) and Sørensen-Dice coefficient (upper triangular part). (D) Same as C, but for individual CR scores obtained from CR weights and subsequent memory coefficients. It is important to note that the CR pattern is multivariate in nature, interpretable as a whole and cluster descriptives are reported for transparency of obtained non-negligible coefficients contributing to the pattern. Source data are provided as a Source Data file. CR = cognitive reserve, PCs = principal components.

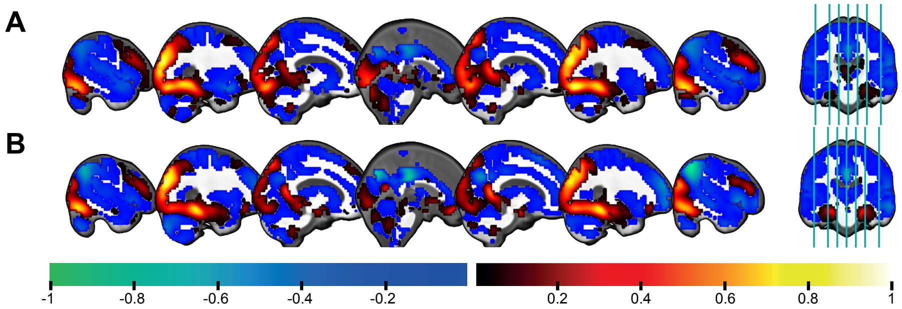


Figure S10: **CR coefficients in whole gray matter.** (A) Results of the same multivariate model when extending the search space to all gray matter instead of only regions contributing to successful memory encoding. (B) Mean beta values of the parametric successful memory contrast. The correlation between A and B is 0.942. All values have been normalized by the highest absolute value of the respective image. Source data are provided as a Source Data file. CR = cognitive reserve.

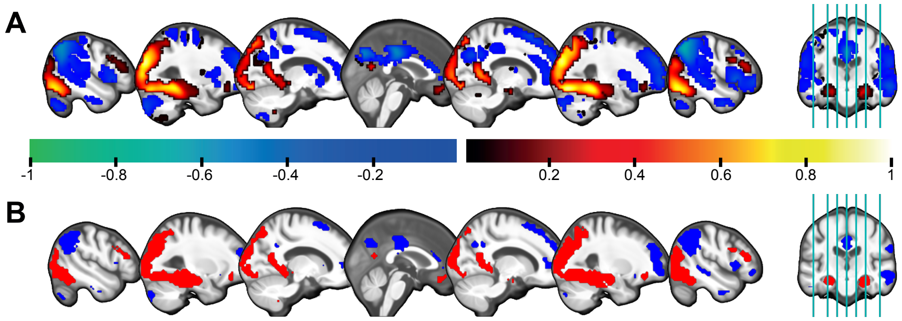


Figure S11: **CR pattern in multivariate model with only 2 principal components.** Results of the same multivariate model using 2 instead of 7 principal components. (A) Voxel-wise moderation coefficients (CR), normalized by the highest absolute coefficient. (B) Regions with significant positive (red) and negative (blue) CR coefficients as indicated by bootstrapping. It is important to note that the CR pattern is multivariate in nature, interpretable as a whole and cluster descriptives are reported for transparency of obtained non-negligible coefficients contributing to the pattern. Source data are provided as a Source Data file. CR = cognitive reserve.

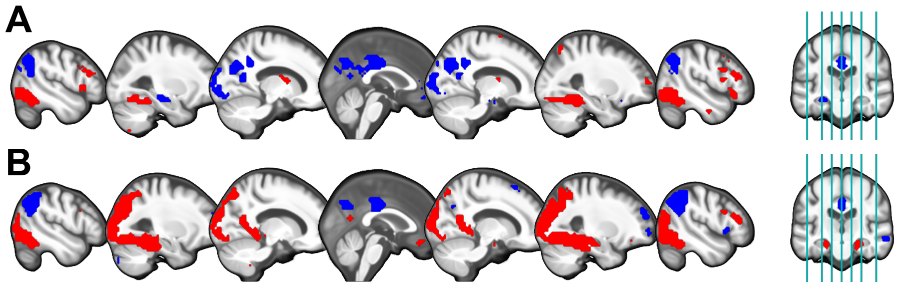


Figure S12: **Significant CR regions according to previous bootstrapping inference approach.** Regions with significant positive (red) and negative (blue) contributions to CR as inferred from a bootstrapping inference approach adopted by Chén et al. (2018) and slightly adjusted. (A) Results for the multivariate model with 7 PCs. (B) Results for the multivariate model with only 2 PCs (compare with Fig. S11 for results with 2 PCs using the updated bootstrap inference method). It is important to note that the CR pattern is multivariate in nature, interpretable as a whole and cluster descriptives are reported for transparency of obtained non-negligible coefficients contributing to the pattern. Source data are provided as a Source Data file. CR = cognitive reserve, PCs = principal components.

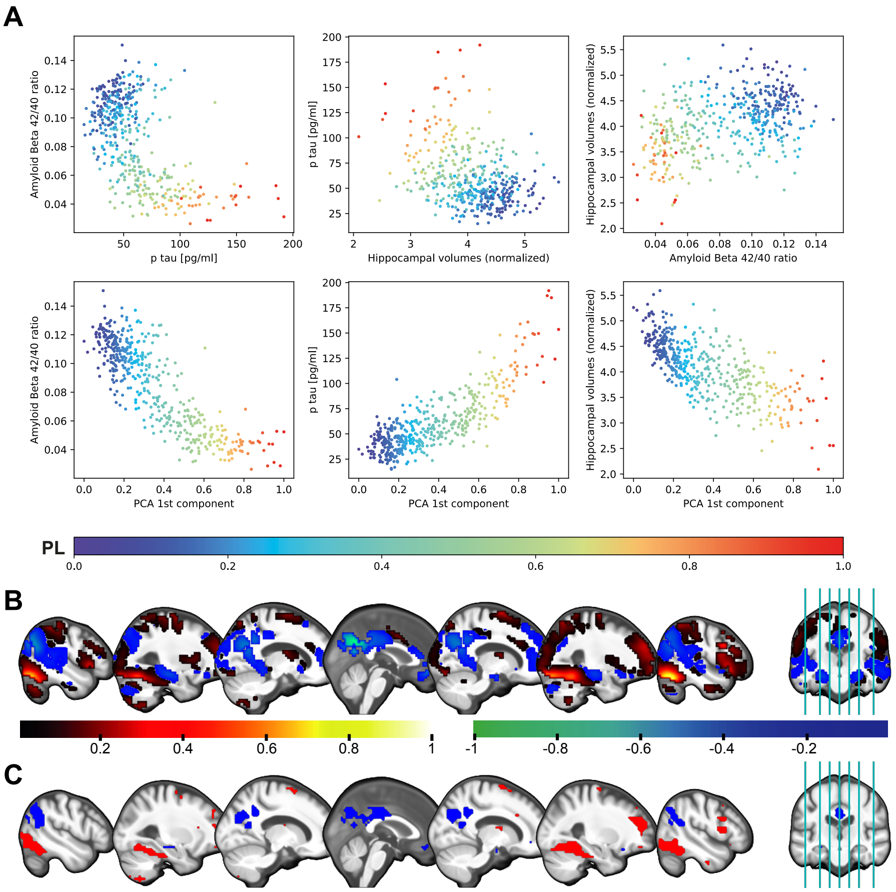


Figure S13: **PCA-based PL score.** Here we present an alternative PL score (r = 0.930 with t-SNE-based PL score) represented by the first component of a PCA on $A\beta_{42:40}$, p-tau and TIV-normalized bilateral hippocampal volumes. (A) Relationship of the PCA-based PL score to AD biomarkers. (B) Voxelwise moderation coefficients (CR) for multivariate model with PCA-based PL score (r = 0.907 with CR coefficients in main text), normalized by the highest absolute coefficient. (C) Regions with significant positive (red) and negative (blue) CR coefficients for multivariate model with PCA-based PL score. It is important to note that the CR pattern is multivariate in nature, interpretable as a whole and cluster descriptives are reported for transparency of obtained non-negligible coefficients contributing to the pattern. Source data are provided as a Source Data file. CR = cognitive reserve, PCA = principal component analysis, PL = pathological load, TIV = total intracranial volume, t-SNE = t-distributed stochastic neighbor embedding.

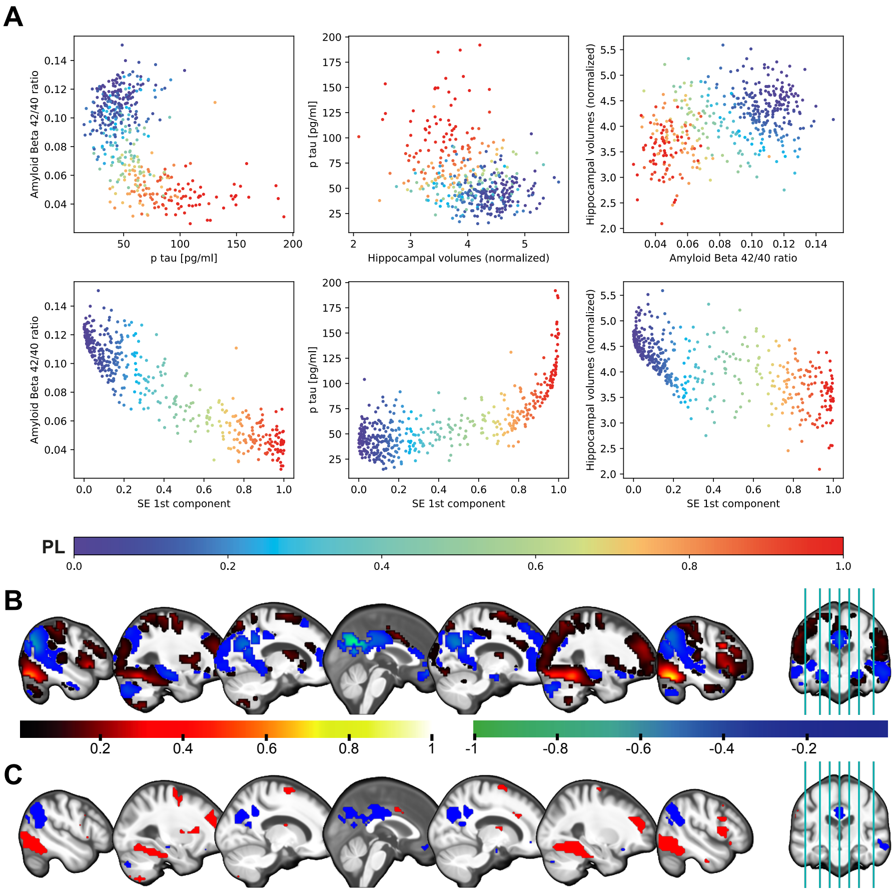


Figure S14: **SE-based PL score.** Here we present an alternative PL score (r = 0.951 with t-SNE-based PL score) obtained as the first component a nonlinear dimensionality reduction method called spectral embedding (SE) on $A\beta_{42:40}$, p-tau and TIV-normalized bilateral hippocampal volumes. (A) Relationship of the SE-based PL score to AD biomarkers. (B) Voxelwise moderation coefficients (CR) for multivariate model with SE-based PL score (r = 0.878 with CR coefficients in main text), normalized by the highest absolute coefficient. (C) Regions with significant positive (red) and negative (blue) CR coefficients for multivariate model with SE-based PL score. It is important to note that the CR pattern is multivariate in nature, interpretable as a whole and cluster descriptives are reported for transparency of obtained non-negligible coefficients contributing to the pattern. Source data are provided as a Source Data file. CR = cognitive reserve, PL = pathological load, SE = spectral embedding, TIV = total intracranial volume, t-SNE = t-distributed stochastic neighbor embedding.

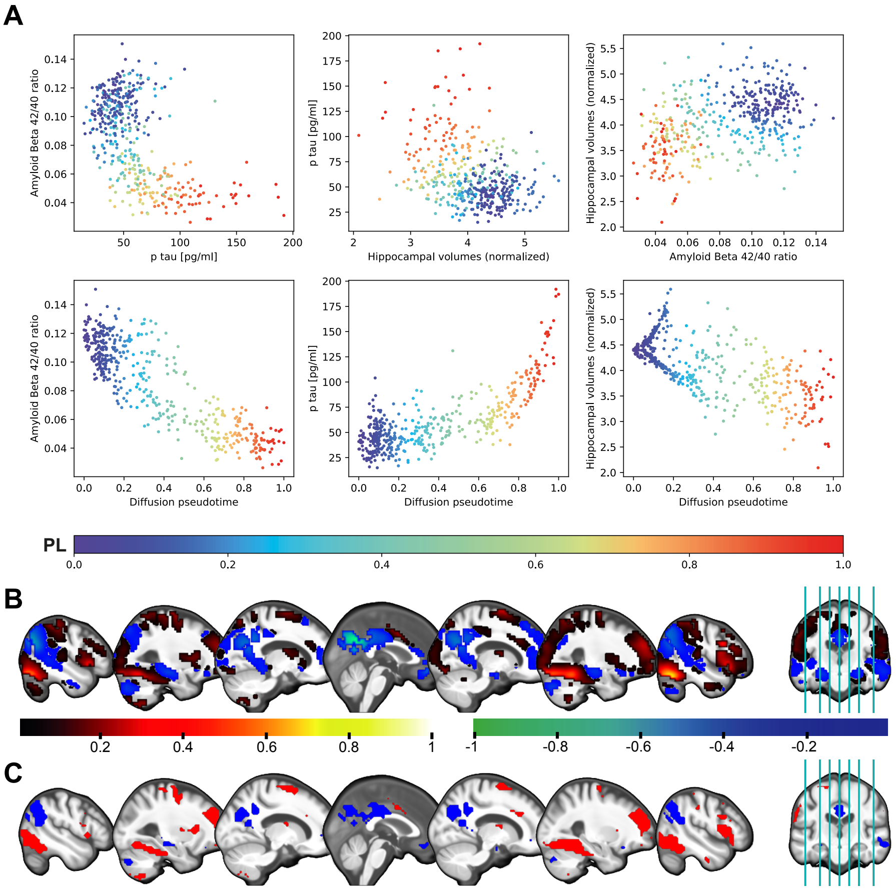


Figure S15: **DPT-based PL score.** Here we present an alternative PL score (r = 0.940 with t-SNE-based PL score) obtained by a diffusion pseudotime (DPT)[15] analysis on $A\beta_{42:40}$, p-tau and TIV-normalized bilateral hippocampal volumes. AD biomarkers of the 26 most pathology-free participants with $A\beta_{42:40} > 0.12$ and p-tau $< 50$ have been averaged and defined as the "root cell" required by the algorithm. (A) Relationship of the DPT-based PL score to AD biomarkers. (B) Voxelwise moderation coefficients (CR) for multivariate model with DPT-based PL score (r = 0.909 with CR coefficients in main text), normalized by the highest absolute coefficient. (C) Regions with significant positive (red) and negative (blue) CR coefficients for multivariate model with DPT-based PL score. It is important to note that the CR pattern is multivariate in nature, interpretable as a whole and cluster descriptives are reported for transparency of obtained non-negligible coefficients contributing to the pattern. Source data are provided as a Source Data file. CR = cognitive reserve, DPT = diffusion pseudotime, PL = pathological load, TIV = total intracranial volume, t-SNE = t-distributed stochastic neighbor embedding.

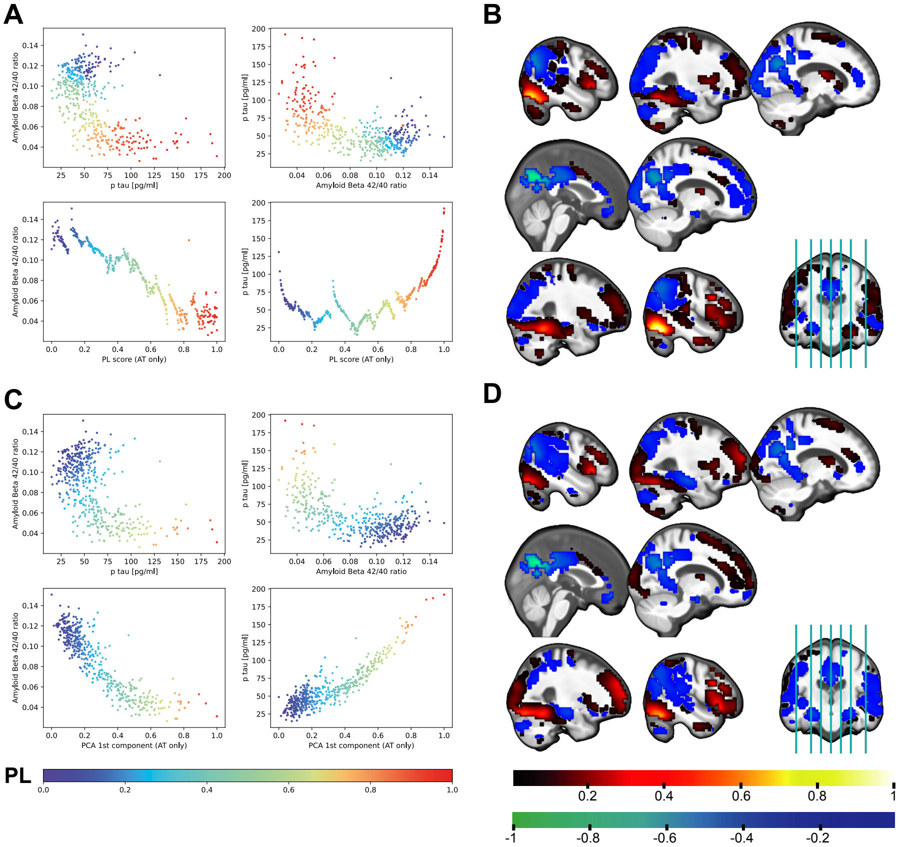


Figure S16: **PL score from A$\beta_{42:40}$ and p-tau only.** Here we present an alternative PL score based only on A$\beta_{42:40}$ and p-tau, excluding hippocampal volumes. (A) Relationship of a t-SNE-based AT PL score with perplexity 25 (r = 0.845 with original PL score) to A$\beta_{42:40}$ and p-tau. (B) Voxelwise moderation coefficients (CR) for multivariate model with t-SNE-based AT PL score (r = 0.961 with CR coefficients in main text), normalized by the highest absolute coefficient. (C) Relationship of a PCA-based AT PL score (r = 0.865 with original PL score) to A$\beta_{42:40}$ and p-tau. (D) Voxelwise moderation coefficients (CR) for multivariate model with PCA-based AT PL score (r = 0.866 with CR coefficients in main text), normalized by the highest absolute coefficient. Color scale below panel C applies to the PL score of both panels A and C. Color scale below panel D applies to the CR coefficients in both panels B and D. Source data are provided as a Source Data file. CR = cognitive reserve, PCA = principal component analysis, PL = pathological load, t-SNE = t-distributed stochastic neighbor embedding.

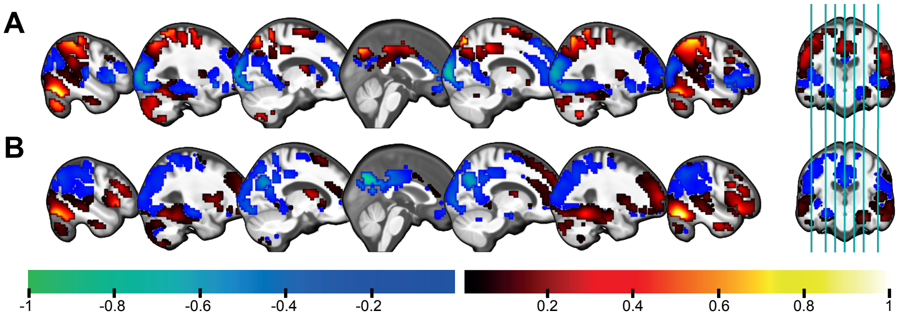


Figure S17: **Separate CR coefficients for CIs and CUs.** (A) Results of the multivariate model when applied only to cognitively impaired people (amnestic mild cognitive impairment, Alzheimer's disease dementia; N = 44; r = 0.150 with CR coefficients in main text). (B) Results of the multivariate model when applying it to only cognitively unimpaired people (cognitively normals, AD patient first-degree relatives, subjective cognitive decliners; N = 184; r = 0.891 with CR coefficients in main text). Please note the substantial difference in sample sizes and many variables between the two groups. The CIs are older (72.52 vs 68.95 years, $p = 2.30 \cdot 10^{-5}$), have higher PL scores (0.637 vs 0.359, $p = 2.52 \cdot 10^{-8}$), lower PACC5 scores (-1.76 vs -0.02, $p = 2.40 \cdot 10^{-12}$) and are less educated (13.36 vs 14.82 years, $p = 5.02 \cdot 10^{-4}$). The latter might be another indication that CR not only may work differently in CIs, but CIs might also simply have lower CR that made them more susceptible to cognitive deficits in the first place. In consequence, they might not be a good model for examining CR. Dedicated samples of cognitively impaired participants might be better suited to examine the neural basis of CR in this special subgroup. CR coefficients were normalized by the highest absolute coefficient, respectively. Source data are provided as a Source Data file. CI = cognitively impaired, CR = cognitive reserve, CU = cognitively unimpaired, PACC5 = Preclinical Alzheimer's Cognitive Composite 5.

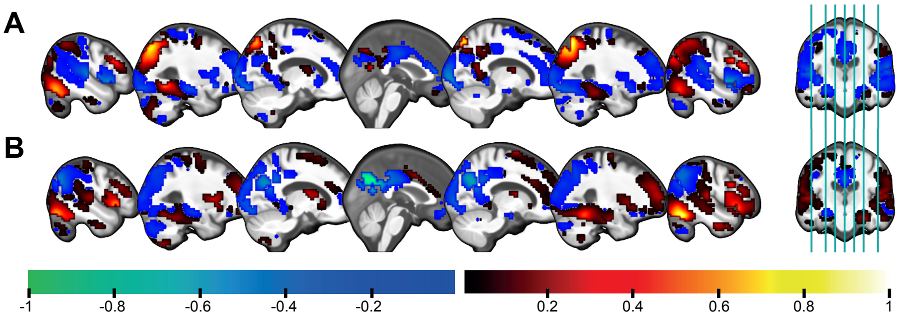


Figure S18: **Separate CR coefficients for females and males.** (A) Results of the multivariate model when applying it to only female participants (N = 114; r = 0.282 with CR coefficients in main text). (B) Results of the multivariate model when applying it to male participants (N = 114; r = 0.879 with CR coefficients in the main text). CR coefficients were normalized by the highest absolute coefficient, respectively. Source data are provided as a Source Data file. CR = cognitive reserve.

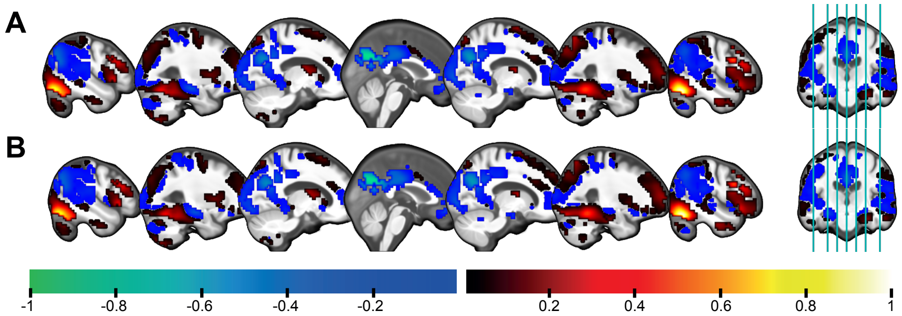


Figure S19: **CR coefficients when accounting for morphometric covariates.** (A) Results of the multivariate model when accounting for mean cortical thickness (from Freesurfer) as an additional covariate (r = 0.999 with CR coefficients in main text). (B) Results of the multivariate model when including mean GM volumes (from SPM segmentation) of the voxels with significant CR contributions (according to the main model) as an additional covariate (r = 0.995 with CR coefficients in the main text). CR coefficients were normalized by the highest absolute coefficient, respectively. Source data are provided as a Source Data file. CR = cognitive reserve.

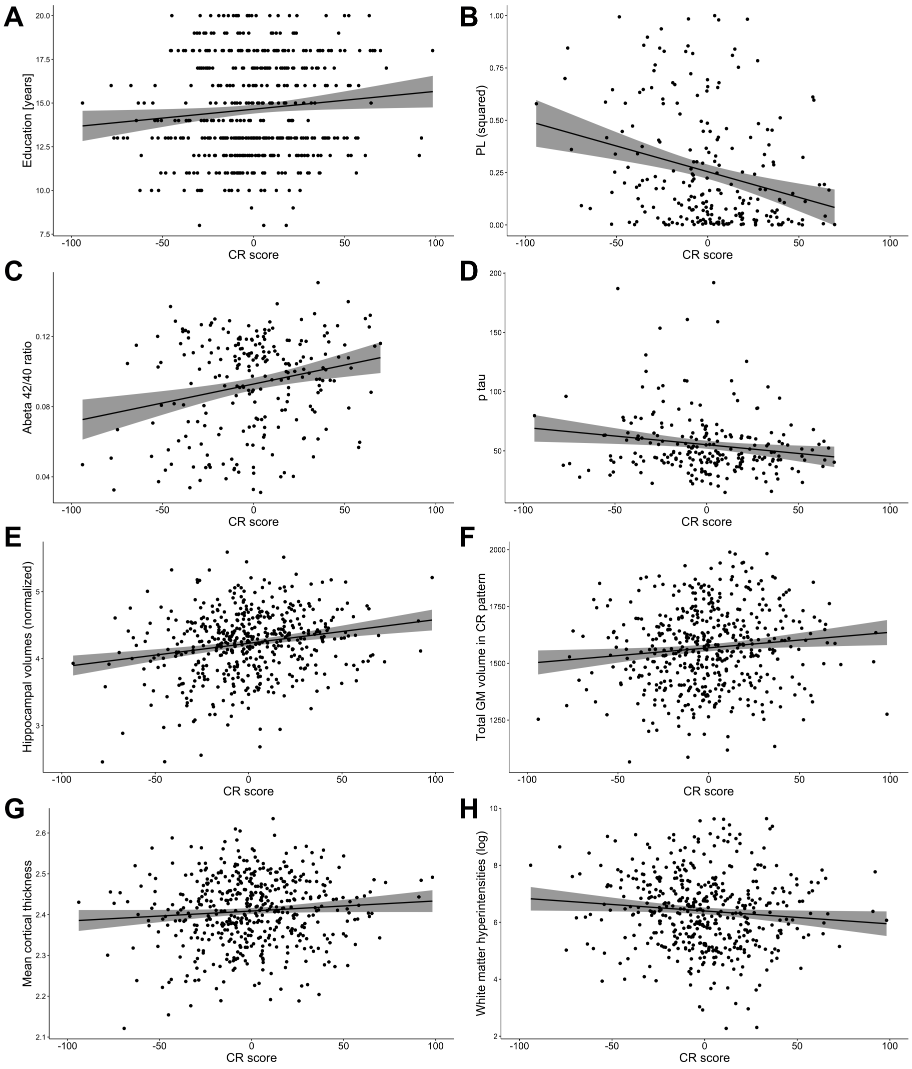


Figure S20: **Correlations of CR score with other variables - part 1.** Scatter plots display the CR score on the x-axis and (A) education, (B) the (squared) PL score, (C) $A\beta_{42:40}$, (D) p-tau, (E) hippocampal volumes (bilaterally averaged and normalized with TIV), (F) sum of GM volumes in the voxels with significant contribution to CR, (G) mean cortical thickness or (H) white matter hyperintensities (log-transformed) on the y-axis. The correlation coefficients and corresponding p values are depicted in Fig. S2. CR = cognitive reserve, GM = gray matter, PL = pathological load, TIV = total intracranial volume.

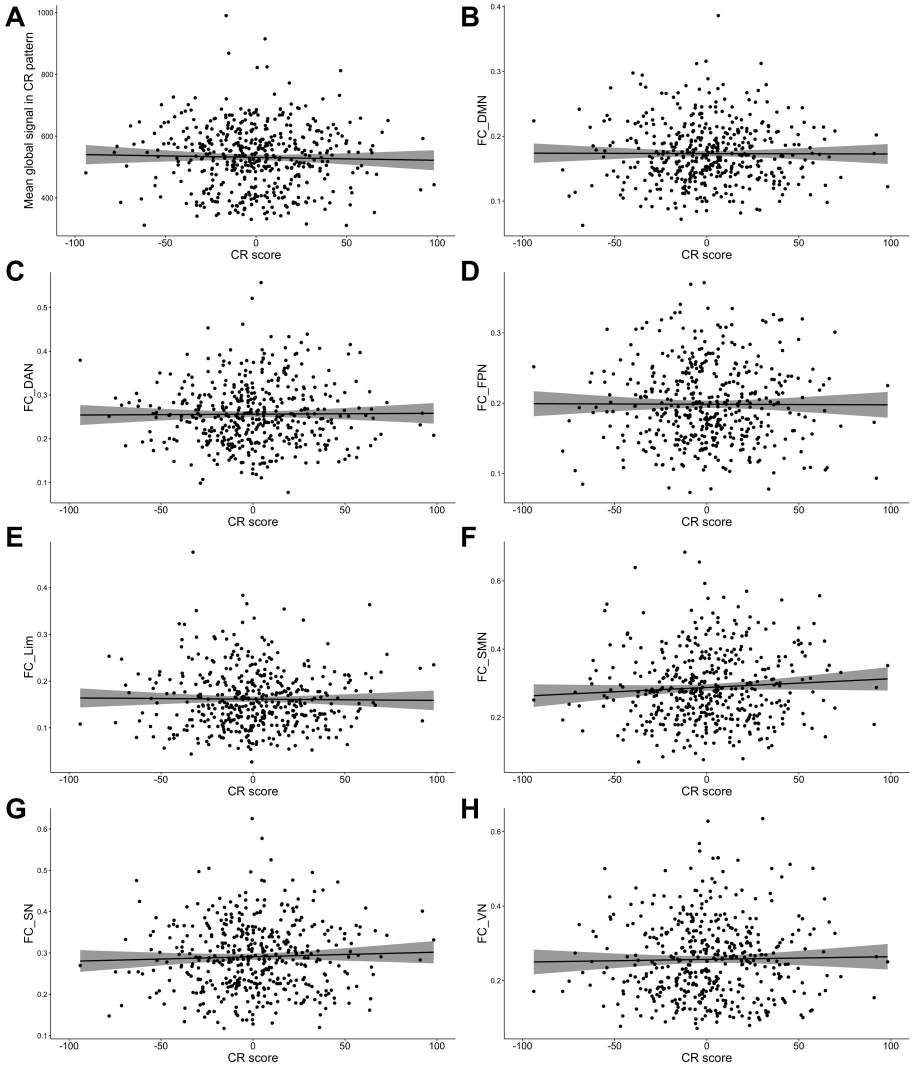


Figure S21: **Correlations of CR score with other variables - part 2.** Scatter plots display the CR score on the x-axis and (A) the mean global signal from resting-state fMRI in voxels with significant contribution to CR, and functional connectivity in the (B) DMN, (C) DAN, (D) FPN, (E) limbic network, (F) SMN, (G) SN and (H) VN on the y-axis. The correlation coefficients and corresponding p values are depicted in Fig. S2. CR = cognitive reserve, DMN = default mode network, DAN = dorsal attention network, FC = functional connectivity, FPN = fronto-parietal network, GM = gray matter, Lim = limbic network, SMN = somatomotor network, SN = salience network, VN = visual network.

## 5.5 Supplementary Tables

Table S2: **Subsample characteristics.** Means ± standard deviations are illustrated. P values were obtained from two-sided two-sample t-test or Chi-square test and not corrected for multiple comparisons. The group contingency of the five diagnostic groups was not significantly different between both subsamples (p = 0.103). Hippocampal volumes were bilaterally averaged and normalized to the total intracranial volume. 4 participants of the CSF subsample and 5 participants of the MRI-only subsample had no Preclinical Alzheimer's Cognitive Composite 5 (PACC5) scores. Source data are provided as a Source Data file.

| | **CSF subsample** | **MRI-only subsample** | **p value** |
|---|---|---|---|
| N | 232 | 258 | |
| Age at baseline [years] | 69.6±5.4 | 69.8±5.9 | 0.713 |
| Sex [% female] | 50.0 | 57.0 | 0.146 |
| Education [years] | 14.6±2.7 | 14.7±3.0 | 0.573 |
| Hippocampal volumes | 4.21±0.53 | 4.25±0.47 | 0.304 |
| PACC5 scores | -0.36±1.05 | -0.05±0.87 | $5.67 \cdot 10^{-4}$ |

Table S3: **Significant clusters in CR activity pattern**. Same as Tab. 2, but includes smaller clusters with sizes between 30 and 50 voxels (all discordant). In two instances, the peak voxel was not contained in the AAL atlas and is thus marked with a question mark. AAL = Automated Anatomic Labeling, CR = cognitive reserve. Source data are provided as a Source Data file.

| # | **Mean $w_i$** | **Size** [voxels] | % **concordant** | **Peak[x,y,z]** | **Peak Structure** |
|---|---|---|---|---|---|
| 8 | 0.056 | 48 | 0 | 35, 59, 7 | Frontal_Mid_R |
| 9 | 0.044 | 45 | 0 | -4, 7, 70 | Supp_Motor_Area_L |
| 10 | 0.053 | 42 | 0 | 42, 14, 39 | Frontal_Inf_Oper_R |
| 11 | 0.058 | 42 | 0 | 7, 3, 73 | Supp_Motor_Area_R |
| 12 | 0.051 | 37 | 0 | 49,14,14 | Frontal_Inf_Oper_R |
| 13 | 0.05 | 36 | 0 | -11,0,10 | ? (Caudate_L/Thalamus_L) |
| 14 | 0.045 | 34 | 0 | -35,-66,-59 | ? (near Cerebellum_L) |
| 15 | 0.043 | 30 | 0 | 49,-7,-32 | Temporal_Inf_R |
| 16 | -0.068 | 30 | 0 | -28,-21,-14 | Hippocampus_L |

Table S4: **Correlation between different PL scores**. All PL scores exhibit very high correlations with each other, but show subtle differences in their patterns of nonlinearity (see Figs. S14, S15, S13). Source data are provided as a Source Data file. DPT = diffusion pseudotime, PCA = principal component analysis, PL = pathological load, SE = spectral embedding, t-SNE = t-distributed stochastic neighbor embedding.

| | t-SNE | PCA | DPT | SE |
|---|---|---|---|---|
| t-SNE | 1.000 | 0.930 | 0.940 | 0.951 |
| PCA | 0.930 | 1.000 | 0.965 | 0.963 |
| DPT | 0.940 | 0.965 | 1.000 | 0.983 |
| SE | 0.951 | 0.963 | 0.983 | 1.000 |

## Supplementary References


[1] Schmidt, P. *et al.* Automated segmentation of changes in FLAIR-hyperintense white matter lesions in multiple sclerosis on serial magnetic resonance imaging. *NeuroImage: Clinical* **23**, 101849 (2019).

[2] Jenkinson, M., Bannister, P., Brady, M. & Smith, S. Improved Optimization for the Robust and Accurate Linear Registration and Motion Correction of Brain Images. *NeuroImage* **17**, 825–841 (2002).

[3] Parkes, L., Fulcher, B., Yücel, M. & Fornito, A. An evaluation of the efficacy, reliability, and sensitivity of motion correction strategies for resting-state functional MRI. *NeuroImage* **171**, 415–436 (2018).

[4] Behzadi, Y., Restom, K., Liau, J. & Liu, T. T. A component based noise correction method (CompCor) for BOLD and perfusion based fMRI. *NeuroImage* **37**, 90–101 (2007).

[5] Friston, K. J., Williams, S., Howard, R., Frackowiak, R. S. & Turner, R. Movement-Related effects in fMRI time-series. *Magnetic Resonance in Medicine* **35**, 346–355 (1996).

[6] Hallquist, M. N., Hwang, K. & Luna, B. The nuisance of nuisance regression: spectral misspecification in a common approach to resting-state fMRI preprocessing reintroduces noise and obscures functional connectivity. *NeuroImage* **82**, 208–225 (2013).

[7] Yeo, B. T. *et al.* The organization of the human cerebral cortex estimated by intrinsic functional connectivity. *Journal of neurophysiology* (2011).

[8] Van Der Maaten, L. & Hinton, G. Visualizing Data using t-SNE. *Journal of Machine Learning Research* **9**, 2579–2605 (2008).

[9] Chén, O. Y. *et al.* High-dimensional multivariate mediation with application to neuroimaging data. *Biostatistics* **19**, 121–136 (2018). `1511.09354`.

[10] Jack, C. R. *et al.* A/T/N: An unbiased descriptive classification scheme for Alzheimer disease biomarkers. *Neurology* **87**, 539–547 (2016).

[11] Düzel, E., Schütze, H., Yonelinas, A. P. & Heinze, H.-J. Functional phenotyping of successful aging in long-term memory: Preserved performance in the absence of neural compensation. *Hippocampus* **21**, n/a–n/a (2010).

[12] Soch, J. *et al.* A comprehensive score reflecting memory-related fMRI activations and deactivations as potential biomarker for neurocognitive aging. *Human Brain Mapping* **42**, 4478–4496 (2021).

[13] Jack, C. R. *et al.* Hypothetical model of dynamic biomarkers of the Alzheimer's pathological cascade. *The Lancet Neurology* **9**, 119–128 (2010).

[14] Vogel, J. W. *et al.* Four distinct trajectories of tau deposition identified in Alzheimer's disease. *Nature medicine* 1–11 (2021).

[15] Haghverdi, L., Büttner, M., Wolf, F. A., Büttner, F. & Theis, F. J. Diffusion pseudotime robustly reconstruct lineage branching. *Nature Methods* (2016).
