# Supplementary material for: Cognitive reserve against Alzheimer’s pathology is linked to brain activity during memory formation
Source: Nat Commun. 2024 Nov 13;15:9815. doi: 10.1038/s41467-024-53360-9 (PMC11561234; doi:10.1038/s41467-024-53360-9)
Supplement: Supplementary file 3 — Reporting Summary [file 41467_2024_53360_MOESM3_ESM.pdf]

nature portfolio

Corresponding author(s): Niklas Vockert
Last updated by author(s): Jul 26, 2024

Reporting Summary

Nature Portfolio wishes to improve the reproducibility of the work that we publish. This form provides structure for consistency and transparency in reporting. For further information on Nature Portfolio policies, see our Editorial Policies and the Editorial Policy Checklist.

Statistics

For all statistical analyses, confirm that the following items are present in the figure legend, table legend, main text, or Methods section.

n/a	Confirmed	
☐	☒	The exact sample size (*n*) for each experimental group/condition, given as a discrete number and unit of measurement
☐	☒	A statement on whether measurements were taken from distinct samples or whether the same sample was measured repeatedly
☐	☒	The statistical test(s) used AND whether they are one- or two-sided<br>*Only common tests should be described solely by name; describe more complex techniques in the Methods section.*
☐	☒	A description of all covariates tested
☐	☐	A description of any assumptions or corrections, such as tests of normality and adjustment for multiple comparisons
☐	☒	A full description of the statistical parameters including central tendency (e.g. means) or other basic estimates (e.g. regression coefficient) AND variation (e.g. standard deviation) or associated estimates of uncertainty (e.g. confidence intervals)
☐	☒	For null hypothesis testing, the test statistic (e.g. *F*, *t*, *r*) with confidence intervals, effect sizes, degrees of freedom and *P* value noted<br>*Give P values as exact values whenever suitable.*
☒	☐	For Bayesian analysis, information on the choice of priors and Markov chain Monte Carlo settings
☒	☐	For hierarchical and complex designs, identification of the appropriate level for tests and full reporting of outcomes
☐	☒	Estimates of effect sizes (e.g. Cohen's *d*, Pearson's *r*), indicating how they were calculated

Our web collection on statistics for biologists contains articles on many of the points above.

Software and code

Policy information about availability of computer code

Data collection	Software "Presentation" (Neurobehavioral Systems Inc.) for stimuli presentation in fMRI and response collection. The exact version number differed between data acquisition sites (14.2, 14.9, 16.2, 16.3, 16.5, 17.1, 18.1).
Data analysis	Python 3.7 for usage of t-SNE algorithm and alternative dimensionality reduction (scikit-learn package) or trajectory inference methods (scanpy package) presented in the supplementary. SPM12 and MatlabR2016b/MatlabR2018a for fMRI data preprocessing and analysis. Custom scripts in Matlab2018a for multivariate moderation model. R 4.2.2 (packages lme4, lmertest and lm.beta) for statistical analyses other than multivariate moderation model.

For manuscripts utilizing custom algorithms or software that are central to the research but not yet described in published literature, software must be made available to editors and reviewers. We strongly encourage code deposition in a community repository (e.g. GitHub). See the Nature Portfolio guidelines for submitting code & software for further information.

Data

Policy information about availability of data

All manuscripts must include a data availability statement. This statement should provide the following information, where applicable:

- Accession codes, unique identifiers, or web links for publicly available datasets
- A description of any restrictions on data availability
- For clinical datasets or third party data, please ensure that the statement adheres to our policy

Data, study protocol, and biomaterials can be shared with partners based on individual data and biomaterial transfer agreements. The code for the multivariate

moderation model can be found on Github under https://github.com/znerp/NI_moderation_mv.

Research involving human participants, their data, or biological material

Policy information about studies with human participants or human data. See also policy information about sex, gender (identity/presentation), and sexual orientation and race, ethnicity and racism.

Reporting on sex and gender	Disaggregated sex and gender data has not been collected. They were not considered in the study design. Sex was self-reported and the distributions were 263 females and 227 males in the analysed sample (whole baseline sample: 550 females, 529 males). Sex and-gender-based analyses were not performed, as the overarching goal of the study was to determine a general fMRI-based pattern of cognitive reserve. Due to a reviewer comment, we conducted a brief additional disaggregated analysis for males and females, respectively, whose results are presented in the supplementary.
Reporting on race, ethnicity, or other socially relevant groupings	No social grouping variables were used.
Population characteristics	See above.
Recruitment	All patient groups (SCD, MCI, AD) were referrals, including self-referrals, to the participating memory centers. The control group and the relatives of AD dementia patients were recruited by standardized public advertisement.
Ethics oversight	The process was led and coordinated by the ethical committee of the medical faculty of the University of Bonn (trial registration number 117/13).

Note that full information on the approval of the study protocol must also be provided in the manuscript.

Field-specific reporting

Please select the one below that is the best fit for your research. If you are not sure, read the appropriate sections before making your selection.

☐ Life sciences ☒ Behavioural & social sciences ☐ Ecological, evolutionary & environmental sciences

For a reference copy of the document with all sections, see nature.com/documents/nr-reporting-summary-flat.pdf

Behavioural & social sciences study design

All studies must disclose on these points even when the disclosure is negative.

Study description	DELCODE is an observational longitudinal memory clinic-based multicenter study in Germany. The analysed data from the study is quantitative.
Research sample	The analysed sample includes 490 participants from the DELCODE study that come from the general German population over 60 years. As the study was particularly designed at investigating subjects with subjective cognitive decline, these are over-represented in the sample. The sample is generally rather low in AD pathology. The sample is well educated with 14.6 years of education (SD: 2.9 years). 53.7% of the participants were female and on average 69.73 years old (SD: 5.6 years). The rationale for using DELCODE data for investigation of cognitive reserve was its apparent suitability, i.e. the availability of a large dataset with a memory encoding-based fMRI task, Alzheimer's Disease biomarkers, and participants that were on a continuum from cognitively normal to at-risk stages for Alzheimer's Disease to cognitively impaired and Alzheimer's Dementia.
Sampling strategy	1079 participants were enrolled into 5 groups at baseline (stratified sampling): subjective cognitive decline (SCD), mild cognitive impairment (MCI), dementia due to Alzheimer's disease (AD), AD patient relatives and cognitively normal controls. The sample size was determined to be powered for the univariate detection of significant predictors of cognitive decline in subjects with SCD. In one multicenter memory clinic study in Europe the frequency of AD type CSF in subjects with SCD was 50%. Due to slight differences in the definition of SCD (i.e. inclusion of subjects reporting worries about other that memory decline in DELCODE) a frequency of 40% individuals in the SCD group that will display evidence for amyloid deposition in the CSF was estimated. One study reported a hazard ratio (HR) of 15 for MCI/dementia (evidenced by episodic memory decline) in memory clinic patients with SCD and Aß42 reduction in the CSF with a mean observation period of 4 years (van Harten et al., 2011). In the present multicenter study, the estimation was more conservative. The assumption for an univariate predictor for episodic memory decline in preclinical AD over 5 years was an odds ratio of 3. With these assumptions (40% SCD subjects with preclinical AD, OR=3, 5 year follow-up, 10% drop-out) 300 patients with SCD are required to identify a predictor of episodic memory decline with 80% power. The sizes of the other groups were defined to be sufficiently large for comparison with the SCD group and to be feasible to recruit within the DZNE multicenter structure.
Data collection	A trained researcher administered the neuropsychological tests (pen and paper). The researchers were not aware of the primary study hypothesis. A researcher recorded the participants' responses in the post-fMRI retrieval task on a computer. The researcher merely recorded the responses and was unaware of the images the subject had seen in the scanner. No one was present except for the researcher and the participant.
Timing	The included data was collected between 2014 and 2021.
Data exclusions	Participants were excluded if they had no fMRI data. 68 of the remaining 558 participants were further excluded based on their behavioral and fMRI data using the following criteria, leading to the final analysed sample of 490 participants:. (1) They made more

	than 8 errors in their indoor/outdoor judgement. This corresponds to individuals with extreme outliers in the distribution of indoor/outdoor errors and could be related to lack of attention or confusion. (2) Based on response bias in their confidence rating during post-MRI retrieval, represented by the criterion location c. Individuals with absolute response bias values above 1.5 were excluded, since strong bias could potentially render the parametric modulation invalid for two reasons. First, the response category would likely not correspond to the actual BOLD signal at the time of encoding. Second, a reliable estimation of the subsequent memory regressor does require some variability in the response categories. (3) Framewise displacement (FD) was above 0.5mm in a single EPI or above 0.2mm in more than 2% of the EPIs. This exclusion was supposed to limit motion effects on the data quality. (4) An individual had extreme outliers in the β values of more than 10% of the voxels of their (GM-masked) regressor image. This was indicative of inaccurate estimations of the subsequent memory regressor in large parts of the brain and could have skewed the results of subsequent modeling steps. The criteria were not pre-established, but empirically informed and used in an attempt to limit the influence of undesired sources of noise.
Non-participation	Decline of participation has not been recorded. 210 participants dropped out of the study for various reasons (119: participant or relative wanted termination; 19: participant is in nursing home; 16: other disease that prevents further participation; 11: decision of the responsible doctor; 7: participant moved away; 6: contact lost; 3: participant is bedridden; 29: other reasons, e.g. death)
Randomization	Participants were not allocated to experimental groups.

Reporting for specific materials, systems and methods

We require information from authors about some types of materials, experimental systems and methods used in many studies. Here, indicate whether each material, system or method listed is relevant to your study. If you are not sure if a list item applies to your research, read the appropriate section before selecting a response.

Materials & experimental systems

n/a	Involved in the study
☒	☐ Antibodies
☒	☐ Eukaryotic cell lines
☒	☐ Palaeontology and archaeology
☒	☐ Animals and other organisms
☒	☐ Clinical data
☒	☐ Dual use research of concern
☒	☐ Plants

Methods

n/a	Involved in the study
☒	☐ ChIP-seq
☒	☐ Flow cytometry
☐	☒ MRI-based neuroimaging

Magnetic resonance imaging

Experimental design

Design type	Task, event-related
Design specifications	132 trials (stimuli) per subject in one session. Each stimulus was presented for 2500ms and the inter-stimulus-interval was jittered around an average of 1250ms with a standard deviation of about 700ms.
Behavioral performance measures	Button press and response time were registered during the task. More than 8 errors in their indoor/outdoor judgement were interpreted as a lack of attention or confusion. Thus, these individuals with extreme outliers in the distribution of indoor/outdoor errors were excluded from the analyses.

Acquisition

Imaging type(s)	Functional and structural
Field strength	3T
Sequence & imaging parameters	T1-weighted image: gradient echo, 3D GRAPPA PAT 2, 1mm^3 isotropic, 256x256 px, 192 slices, sagittal, ca. 5min, TR 2500ms, TE 4.33ms, TI 1100ms, FA 7 degrees.<br>T2-weighted (optimized for medial temporal lobe volumetry): spin echo, 0.5x0.5x1.5 mm^3, 384x384 px, 64 slices, orthogonal to the hippocampal long axis, ca. 12min, TR 3500ms, TE 353ms. SE<br>task fMRI: gradient echo, 2D EPI, GRAPPA PAT 2, 3.5mm^3 isotropic, 64x64px, 47 slices, oblique axial/AC-PC aligned, ca. 9 min, TR 2580ms, TE 30ms, FA 80 degrees, 206 volumes.
Area of acquisition	T1-weighted and fMRI were whole-brain, the T2-weighted scan covered the medial temporal lobe region. The region was determined visually on the T1-weighted scans.

Diffusion MRI ☐ Used ☒ Not used

Preprocessing

Preprocessing software	SPM12 in Matlab2016b
Normalization	Non-linear transformation to MNI space.
Normalization template	MNI152
Noise and artifact removal	Unwarping of the functional images was done using voxel-displacement maps derived from fieldmaps to correct for distortions of the images. Exclusion of images with framewise displacement above 0.5mm in a single EPI or above 0.2mm in more than 2% of the EPIs.
Volume censoring	No volume censoring was performed.

Statistical modeling & inference

Model type and settings	Multivariate moderation model with custom code for the second level. The model used cognitive performance (Box-Cox transformed PACC5 values) as an outcome variable. Predictor variables were pathological load (squared), seven principal components of the first-level contrast images and their interactions with pathological load. Further, the covariates age at baseline, sex, total intracranial volume and MR acquisition site entered the model. See formula in methods section (4.7).
Effect(s) tested	The coefficients of the interaction terms of the principal components were projected back into the original image space to assign each examined voxel a moderation coefficient (representing cognitive reserve). Inference on the moderation coefficients was performed using a bootstrapping approach (see below).

Specify type of analysis: ☐ Whole brain ☒ ROI-based ☐ Both

Anatomical location(s)	Regions of interest were selected based on a second-level analysis of the first-level subsequent memory contrast images. Voxels with a beta value statistically different from 0 (FWE-corrected $p < 0.05$) were considered part of the regions of interest.

Statistic type for inference (See Eklund et al. 2016)	Voxel-wise inference with a bootstrapping approach. See methods section (4.8.2.) for details.
Correction	No explicit correction for multiple comparisons due to the multivariate nature of the approach. In contrast to a mass-univariate approach, only one model is fitted for all voxels instead of one per voxel.

Models & analysis

n/a	Involved in the study
☒	☐ Functional and/or effective connectivity
☒	☐ Graph analysis
☐	☒ Multivariate modeling or predictive analysis

Multivariate modeling and predictive analysis	t-SNE was used for dimensionality reduction of the three variables CSF Abeta42/40, CSF p tau and hippocampal volumes (corrected for total intracranial volumes) to a single variable of pathological load, which was used as an independent variable. Likewise, the first-level contrast images were reduced to seven variables using principal component analysis and used as independent variables. The dimensionality reduction via t-SNE was only checked for robustness with 1000 repetitions. The optimal number of principal components was determined in a 10-fold cross-validation approach, which was repeated 10 times to ensure different partitioning of the data into folds. The training data was used to obtain coefficients for the multivariate moderation model in predicting cognitive performance. With these coefficients the held-out (test) data was predicted. Across the ten folds all data was predicted once based on the remaining 90% for each number of principal components. The coefficient of determination (R^2) between the true and predicted PACC5 values (Box-Cox transformed) was calculated based on the aggregated data, done once per number of principal components. The optimal number of principal components was identified as the corresponding model with the highest mean R^2 value across the 10 predictions.
